# Multiple hepatocellular adenomas associated with long-term administration of androgenic steroids for aplastic anemia: A case report and literature review

**DOI:** 10.1097/MD.0000000000020829

**Published:** 2020-07-10

**Authors:** Lixia Wang, Cong Wang, Wei Li, Fanyang Meng, Yuying Li, Hongqiong Fan, Yanhua Zhou, Gnana Bharathi, Sujun Gao, Yan Yang

**Affiliations:** a Department of Hematology; b Department of Radiology, The First Hospital of Jilin University; c Department of Hematology, the Qianwei Hospital of Jilin Province, Changchun, Jilin, China.

**Keywords:** anabolic androgenic steroid, aplastic anemia, hepatocellular adenoma ;

## Abstract

**Introduction:**

Anabolic steroids are widely administered to patients with aplastic anemia (AA) and are associated with numerous medical complications. To assist with future diagnoses, we report about a young boy with multiple hepatocellular adenomas (HAs) induced by long-term use of anabolic androgenic steroids (AAS) for AA and present a related literature review.

**Patient concern:**

A 15-year-old boy who was diagnosed with AA in 2011 had been treated with stanozolol (6 mg per day) and ciclosporin A (120–150 mg per day) for almost 4 years. He presented with epigastric pain and fever, and abdominal computed tomography showed a lesion of heterogenous density measuring 13.5 × 13.0 × 8.0 cm in the left hepatic lobe, which was initially misdiagnosed as a liver abscess.

**Diagnosis:**

The patient went into hemorrhagic shock twice after invasive manipulation that aimed at diagnosis and was finally diagnosed with HA using fine needle aspiration.

**Interventions:**

The patient discontinued AAS and only reserved ciclosporin A for AA treatment.

**Outcomes:**

Follow-up abdominal computed tomography performed 4 years after AAS discontinuation showed obvious regression of the hepatic lesions.

**Conclusion:**

It is of great importance for hematologists to completely understand that the long-term use of AAS may cause HA, which carries a great risk of hemorrhage and malignant transformation.

## 1 Introduction

Anabolic androgenic steroids (AAS) are widely used by bodybuilders to achieve a rapid increase in muscle mass and by aplastic anemia (AA) patients to stimulate hematopoiesis. A growing number of reports suggest that the abuse of AAS is associated with serious adverse effects. Several liver disorders have been reported to be related to AAS administration, namely cholestatic jaundice, peliosis hepatis, hepatocellular adenoma (HA), and hepatocellular carcinoma.^[1]^ HA is an uncommon benign epithelial liver tumor with high risk of malignant transformation, spontaneous hemorrhage, and rupture.^[2–4]^ As a result, early detection is important in order to avoid these associated life-threatening conditions. Here, we report a case of AAS-induced HA who suffered hemorrhagic shock because of an invasive diagnostic operation. Our aim is to share our lessons with the wider medical field and highlight the diagnosis and treatment of AAS-induced HA.

## 2 Case presentation

**Figure 1:**
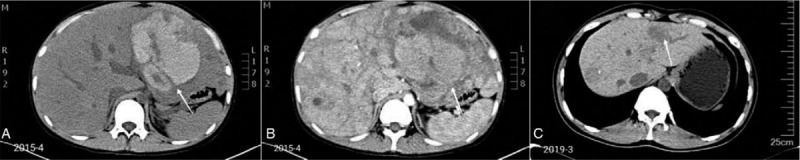
A. A heterogeneous density lesion measuring 13.0 × 8.0 × 13.5 cm was observed in the left hepatic lobe, with an unclear margin. The lesion had uneven density in areas of high central density surrounded by irregular low density. CT values ranged from 15 to 64 HU. B. Dynamic contrast study revealed no enhancement of the lesion. There were another two lesions in the left lateral hepatic lobe and right posterior lobe, measuring 3.4 × 2.2 cm and 2.0 × 1.5 cm, respectively. In contrast, the enhanced arterial phase showed multiple diffuse nodules which were significantly reinforced. The degree of enhancement was in decreasing order in the portal venous phase and the delayed phase. There was no intrahepatic bile duct expansion. C. Low-density lesion measuring 2.0 × 3.0 cm was seen in in left hepatic lobe. There was a patchy slightly high density shadow in the lesion. CT values ranged from 35 to 42 HU.

A 15-year-old boy presented to the hepatic surgery department of our hospital with abdominal pain, accompanied by fever (39.0 °C) in 2015. He was diagnosed with non-severe AA in 2011 and had been taking stanozolol (6 mg per day) and ciclosporin A (120–150 mg per day) for almost 4 years to treat the condition. Physical examination disclosed tenderness in the left upper quadrant of the abdomen, obvious hepatomegaly, and mild jaundice. A routine blood test showed a white blood cell (WBC) count of 9.69 × 10^9^/L (reference range: 3.5–9.5 × 10^9^/L), a neutrophil absolute value (NE#) of 6.71 × 10^9^/L (1.8–6.3 × 10^9^/L), a hemoglobin (HGB) level of 61 g/L (130–175 g/L), and a platelet (PLT) count of 25 × 10^9^/L (125–350 × 10^9^/L). Liver function examinations were notable for elevated levels of aspartate aminotransferase (AST) at 134.0 U/L (13–35 U/L), alanine aminotransferase (ALT) at 142.7 U/L (7–40 U/L), γ-glutamyltranspeptidases (γ-GT) at 96.6 U/L (7–45 U/L), alkaline phosphatase (ALP) at 3402 U/L (50–135 U/L), total bilirubin at 55.9 umol/L (6.8–30 umol/L), and direct bilirubin at 32.0 umol/L (0–8.6 umol/L). Coagulation tests were entirely normal, as were serum levels of alpha-fetoprotein (AFP). Hepatitis virus markers, including hepatitis B and C, were negative. Computed tomography (CT) of the abdomen showed a lesion of heterogeneous density measuring 13.5 × 13.0 × 8.0 cm in the left hepatic lobe (Fig. 1A). The primary diagnosis was made as a liver abscess; ultrasonic-guided percutaneous hepatic drainage was then applied, draining 2000 ml of fresh blood and resulting in hemorrhagic shock. The patient was no longer in danger after extracting the drainage tube, administering hemostatic drugs, and undergoing erythrocyte transfusion 2 days later. His temperature was controlled by administering antibiotics for half a month, but the liver mass did not shrink.

The patient was then transferred to the hematologic department to determine the exact diagnosis. Following this, a 3-phase enhanced CT scan revealed that the liver was significantly enlarged. A lesion of heterogeneous density measuring 13.5 × 13.0 × 8.0 cm was observed in the left hepatic lobe, though the margin was less clear. There were another two lesions in the left lateral hepatic lobe and the right posterior lobe, measuring 3.4 × 2.2 cm and 2.0 × 1.5 cm, respectively. Dynamic contrast study revealed no enhancement of the lesions (Fig. 1B). Cytology was then performed by fine needle aspiration (FNA) of the left nodule. However, soon after this invasive manipulation, the patient underwent hemorrhagic shock again because the tumor was abundant with vessels and prone to bleeding into the peritoneal cavity. The active bleeding was ceased and the patient's vital signs stabilized 3 days later, after positive therapy. The FNA sample did not reveal any malignancy, with immunohistochemical results indicating CD34 (+), CK7(−), GPC-3(−), and β-catentin (+), finally confirming the diagnosis as HA. These multiple HAs, which were distributed diffusedly in the liver, might have been induced by the long-term administration of AAS. Therefore, we recommended cessation of stanozolol consumption to the patient, and to check the hepatic lesions every 6 months. The abdomen symptoms never returned, and the hepatic lesions gradually regressed according to ultrasound (US) examinations. After discontinuation of AAS for 4 years, a final CT scan showed a low density lesion measuring 3.0 × 2.0 cm in the left hepatic lobe, which had obviously regressed (Fig. 1C).

## 3 Discussion

### 3.1 Clinical features of HA

HAs are typically solitary lesions (70%–80%) that range in size from a few millimeters to thirty centimeters, while AAS-induced HAs appear to predispose multiple lesions.^[5]^ There is usually no fibrous capsule, and as a result, hemorrhage from an adenoma can freely extend into the liver and even into the peritoneal cavity.^[6]^ The presence of abdominal pain, a history of extended AAS use, the subcapsular location, and an adenoma size ≥35 mm are all features associated with an increased risk of intra-abdominal bleeding.^[7]^ As for the physical examination of HA, an abdominal lump can be identified in up to 30% of patients, while hepatomegaly is present in approximately 25%. Jaundice has also been described during presentation and presumably reflects compression of the intrahepatic bile ducts by an enlarging mass.^[8,9]^

In this case, the patient presented with epigastric pain and fever, while physical examination revealed obvious hepatomegaly and mild jaundice. During the diagnostic process of this case, we did not fully realize the hemorrhage risk of the tumor, resulting in the patient experiencing hemorrhagic shock twice; as these events were life-threatening, we must draw attention to this condition to raise the awareness of other hematologists.

### 3.2 Association between AAS and HA

In recent years, AAS have been proven to be involved in the development of HAs for the treatment of AA, hereditary angioedema (HAE), and muscle mass development in bodybuilding and transgender individuals.^[10–16]^ A recent study enrolled 182 individuals who used AAS for ≥6 months and found a broad spectrum of liver injuries in this cohort, including hepatotoxicity (46/182) and HA (1/182).^[17]^ Although AAS-induced HAs are relatively rare, the possibility that oral AAS can induce liver cell proliferation must be taken into consideration.

We summarized 11 recently published cases of AAS-induced HAs in Table 1. The median age of these patients was 32 years old (range: 20–69), with a male/female ratio of 1.2:1 (6/5). Because many young men are body-builders, this group in particular is susceptible to AAS-induced HA. The median time from AAS intake to HA onset was 13 years (range: 0.5–20); and for that AA patient the time duration is 6 years which is similar with our case. Most of the AAS-induced HAs were multiple lesions, and the diameter of these lesions was usually greater than 5 cm.

**Table 1:**
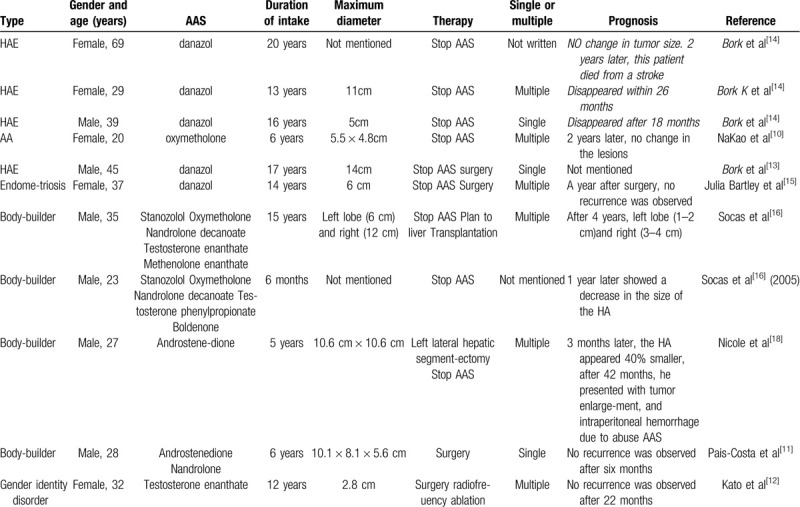
Summarized features of previous case reports.

The possibility of AAS-induced HA cannot be ruled out in our case for the following reasons. Several studies have reported stanozolol as a trigger of HA, and this patient had a 4-year history of exposure to stanozolol before the onset of HA. The clinical characteristics of this patient were consistent with those detailed in the literature; he was an adolescent male who was susceptible to developing AAS-induced HAs, and the multiple lesions seen in his liver are common indicators of AAS-induced HA. Most importantly, the lesions were significantly regressed after AAS withdrawal.

### 3.3 Diagnosis of HA

Since biochemical test abnormalities are rare in HA, multiple imaging modalities are of great importance for diagnosis, including US, CT, and magnetic resonance imaging (MRI) of the abdomen. HA is a vascular tumor with a predominantly arterial supply in classic cases. Expected findings consist of well-vascularized and well-defined solid lesions that are predominantly in an arterial phase on CT or MRI. As seen in our case, the lesions may be dyshomogeneous, especially if hemorrhage, necrosis, or fibrosis is present.^[5,19]^

Dynamic MRI with a hepatocyte-specific contrast agent, such as gadobenate dimeglumine, is the best modality for diagnosing HAs. The tumor can have a clearly defined central margin with nearly parallel vessels entering from the periphery, giving the appearance of a spoked wheel.^[20]^ The sensitivity and specificity of MRI for HA may be as high as 88% to 100%.^[21]^ Compared with conventional MRI, three-phase hepatobiliary MRI with delayed images has a specificity of 100% and a high sensitivity, allowing for the accurate diagnosis of HA; it is particularly valuable for HA lesions smaller than 3 cm.^[22]^

Contrast-enhanced ultrasound (CEUS) is a rising imaging technique that is increasingly used to diagnose liver lesions, as it is important for differentiating between HA and focal nodular hyperplasia (FNH).^[23]^ Additionally, different molecular subtypes of HA have particular B-mode echogenicity contrast-enhanced imaging appearances, and so can be complement approaches to MRI.^[24]^

Percutaneous liver biopsy or FNA are usually not utilized for diagnosis because HA has a tendency to bleed following biopsy, and because the amount of tissue obtained using these methods is frequently insufficient for establishing a diagnosis.^[25,26]^ In the present case, FNA was an important procedure for confirming the HA diagnosis, but also resulted in intra-abdominal bleeding and hemorrhagic shock. Therefore, we learned that multiple HAs can be seen in AA patients who use AAS over an extended period, and FNA should not be recommended for diagnosis as life-threatening complications may occur.

### 3.4 Treatment and prognosis of HA

Treatment of HA mainly depends on symptoms, size, number of lesions, location, and risks of hemorrhage and malignant transformation, and include cessation of associated drugs, surgical resection, transarterial embolization (TAE), thermal ablation, and liver transplantation.

Conservative management may be preferred if the HA is small (<5 cm) and associated with oral AAS.^[26]^ In case of AAS-induced HA, discontinuation of AAS may regress the mass and thereby potentially prevent unnecessary hepatic surgery. Martijn et al^[27]^ stated that 98% of HAs follow this trend, and a conservative approach could lead to HA regression below 50 mm. Moreover, a study of 180 patients diagnosed with HA >5 cm found that 81 patients (45%) treated with conservative therapy reached the clinical regression endpoint of <5 cm after a median of 34 months.^[28]^ In the cases summarized in Table 1, HAs either remain stable or show obvious regression after AAS cessation. In our case, the patient was diagnosed with AAS-induced HA and his left hepatic lobe also regressed from 13.5 cm to 3 cm after cessation of AAS for 4 years.

TAE is considered as the first choice in the management of hemodynamically stable patients with bleeding HA.^[29]^ In the elective setting, TAE has also been performed as an alternative to surgical intervention. In a systematic review of 851 patients with HA, 151 patients (17.7%) underwent TAE with a reported tumor regression rate of 75%. Complete tumor disappearance was observed in 10% of patients and surgery was avoided in 45% of patients.^[30]^ Thermal ablation is a common minimally invasive alternative to surgery which has been recently reported as a treatment method for HA.^[31,32]^ While current studies have only involved a limited number of patients, ablative methods have typically demonstrated efficacy for small HAs (<5 cm).

However, even after the discontinuation of AAS, growth, rupture, and malignant transformation have all been documented.^[33]^ Therefore, HAs that do not resolve after cessation of AAS should also be considered for surgical resection. One controversial issue in the surgical management of HA is the timing of resection after cessation of AAS therapy. Liver transplantation should be reserved for patients for whom surgical resection is not possible due to tumor size/location and for those with adenomatosis.^[34]^

Moreover, male gender, tumor size, and AAS-induced HA are known risk factors for malignant transformation among patients with HA.^[35]^ Although the hepatic lesions of our patient were regressed obviously after AAS cessation, there is still a high risk of malignant transformation; therefore, a careful follow up of our patient by imaging methods and AFP every 6 months is necessary for continued management.

## 4 Conclusion

AA patients, especially those taking AAS over an extended period, should be considered at risk of developing HA. Multiple imaging modalities are considered to be the most important methods for diagnosis, while FNA should be used with caution as life-threatening bleeding complications may occur. If HA is diagnosed, or even suspected, the use of AAS should be discontinued as soon as possible and the appropriate treatment strategy should be carefully selected according to the individual's condition. Because of the high risk for malignant transformation, patients should also be monitored carefully with biochemical analyses of liver function, AFP serum levels, and US studies.

## Acknowledgments

We are grateful to the patient and his family, who gave his informed consent for publication.

## Author contributions

**Investigation:** Yuying Li,Hongqiong Fan, Yanhua Zhou,. Gnana Bharathi.

**Radiologic imagines:** Fanyang Meng.

**Resources:** Wei Li.

**Writing – original draft:** Lixia Wang, Cong Wang.

**Writing – review & editing:** Sujun Gao, Yan Yang.

## References

[1] Büttner A Thieme D Side effects of anabolic androgenic steroids: pathological findings and structure-activity relationships. Handb Exp Pharmacol 2010 459–84. 20020376 10.1007/978-3-540-79088-4_19

[2] Nault JC Couchy G Balabaud C Molecular classification of hepatocellular adenoma associates with risk factors, bleeding, and malignant transformation. Gastroenterology 2017;152:880–94. 27939373 10.1053/j.gastro.2016.11.042

[3] Bossen L Grã¸Nbaek H Lykke EP Men with biopsy-confirmed hepatocellular adenoma have a high risk of progression to hepatocellular carcinoma: a nationwide population-based study. Liver Int 2017;37:1042–6. 28317318 10.1111/liv.13423

[4] European Association for the Study of the Liver (EASL). EASL Clinical Practice Guidelines on the management of benign liver tumours. J Hepatol 2016;65:386–98. 27085809 10.1016/j.jhep.2016.04.001

[5] Grazioli L Federle MP Brancatelli G Hepatic adenomas: imaging and pathologic findings. Radiographics 2001;21:877. 11452062 10.1148/radiographics.21.4.g01jl04877

[6] Minami Y Kudo M Kawasaki T Intrahepatic huge hematoma due to rupture of small hepatocellular adenoma: a case report. Hepatol Res 2002;23:145–51. 12048069 10.1016/s1386-6346(01)00164-4

[7] Bieze M Phoa SS Verheij J Risk factors for bleeding in hepatocellular adenoma. Br J Surg 2014;101:847–55. 24760723 10.1002/bjs.9493

[8] Sturvetant F Moghissi K Oral contraceptives and liver tumors. Controversies in contraception. Baltimore: Williams and Wilkins; 1979 93.

[9] Neuberger J Davis M Williams R Davis M Tredger J Williams R Clinical aspects of oral contraceptive-associated liver tumors. Drug reactions and the liver. Bath, UK: Pittman Medical; 1981 271.

[10] Nakao A Sakagami K Nakata Y Multiple hepatic adenomas caused by long-term administration of androgenic steroids for aplastic anemia in association with familial adenomatous polyposis. J Gastroenterol 2000;35:557–62. 10905366 10.1007/s005350070081

[11] Pais-Costa SR Lima OA Soares AF Giant hepatic adenoma associated with anabolic-androgenic steroid abuse: case report. Arq Bras Cir Dig 2012;25:180–2. 23411809 10.1590/s0102-67202012000300010

[12] Kato K Abe H Hanawa N Hepatocellular adenoma in a woman who was undergoing testosterone treatment for gender identity disorder. Clin J Gastroenterol 2018;11:401–10. 29589251 10.1007/s12328-018-0854-4

[13] Bork K Schneiders V Danazol-induced hepatocellular adenoma in patients with hereditary angio oedema. J Hepatol 2002;36:707–9. 11983460 10.1016/s0168-8278(02)00035-1

[14] Bork K Pitton M Harten P Hepatocellular adenomas in patients taking danazol for hereditary angio-oedema. Lancet (London, England) 1999;353:1066–7. 10.1016/S0140-6736(99)00110-5 10199359

[15] Bartley J Loddenkemper C Lange J Hepatocellular adenoma and focal nodular hyperplasia after long-term use of danazol for endometriosis: a case report. Arch Gynecol Obstet 2004;269:290–3. 15221322 10.1007/s00404-002-0435-z

[16] Socas L Zumbado M Perez-Luzardo O Hepatocellular adenomas associated with anabolic androgenic steroid abuse in bodybuilders: a report of two cases and a review of the literature. Br J Sports Med 2005;39:e27. 15849280 10.1136/bjsm.2004.013599 PMC1725213

[17] Schwingel PA Cotrim HP Santos CR Recreational anabolic-androgenic steroid use associated with liver injuries among Brazilian young men. Subst Use Misuse 2015;50:1490–8. 26549387 10.3109/10826084.2015.1018550

[18] Martin NM Abu Dayyeh BK Chung RT Anabolic steroid abuse causing recurrent hepatic adenomas and hemorrhage. World J Gastroenterol 2008;14:4573–5. 18680242 10.3748/wjg.14.4573 PMC2731289

[19] Choi BY Nguyen MH The diagnosis and management of benign hepatic tumors. J Clin Gastroenterol 2005;39:401–12. 15815209 10.1097/01.mcg.0000159226.63037.a2

[20] Shreenath AP Kahloon A Hepatic (Hepatocellular) Adenoma. StatPearls. Treasure Island (FL): StatPearls Publishing LLC; 2019. 30020636

[21] Ronot M Bahrami S Calderaro J Hepatocellular adenomas: accuracy of magnetic resonance imaging and liver biopsy in subtype classification. Hepatology (Baltimore, Md) 2011;53:1182–91. 10.1002/hep.24147 21480324

[22] Roux M Pigneur F Baranes L Differentiating focal nodular hyperplasia from hepatocellular adenoma: is hepatobiliary phase MRI (HBP-MRI) using linear gadolinium chelates always useful? Abdom Radiol (New York) 2018;43:1670–81. 10.1007/s00261-017-1377-z 29110059

[23] Taimr P Broker MEE Dwarkasing RS A model-based prediction of the probability of hepatocellular adenoma and focal nodular hyperplasia based on characteristics on contrast-enhanced ultrasound. Ultrasound Med Biol 2017;43:2144–50. 28743375 10.1016/j.ultrasmedbio.2017.05.011

[24] Dietrich CF Tannapfel A Jang HJ Ultrasound imaging of hepatocellular adenoma using the new histology classification. Ultrasound Med Biol 2019;45:1–0. 30396597 10.1016/j.ultrasmedbio.2018.06.015

[25] Sandonato L Cipolla C Graceffa G Giant hepatocellular adenoma as cause of severe abdominal pain: a case report. J Med Case Rep 2007;1:57. 17662116 10.1186/1752-1947-1-57 PMC1950307

[26] Terkivatan T de Wilt JH de Man RA Indications and long-term outcome of treatment for benign hepatic tumors: a critical appraisal. Arch Surg 2001;136:1033–8. 11529826 10.1001/archsurg.136.9.1033

[27] Haring MPD Gouw ASH de Haas RJ The effect of oral contraceptive pill cessation on hepatocellular adenoma diameter: a retrospective cohort study. Liver Int 2019;39:905–13. 30773766 10.1111/liv.14074 PMC6593966

[28] Klompenhouwer AJ Alblas M van Rosmalen BV Development and validation of a model to predict regression of large size hepatocellular adenoma. Am J Gastroenterol 2019;114:1292–8. 30920416 10.14309/ajg.0000000000000182

[29] Agrawal S Agarwal S Arnason T Management of hepatocellular adenoma: recent advances. Clin Gastroenterol Hepatol 2015;13:1221–30. 24909909 10.1016/j.cgh.2014.05.023

[30] van Rosmalen BV Coelen RJS Bieze M Systematic review of transarterial embolization for hepatocellular adenomas. Br J Surg 2017;104:823–35. 28518415 10.1002/bjs.10547

[31] Mironov O Jaberi A Beecroft R Retrospective single-arm cohort study of patients with hepatocellular adenomas treated with percutaneous thermal ablation. Cardiovasc Intervent Radiol 2018;41:935–41. 29417268 10.1007/s00270-018-1893-4

[32] Smolock AR Cristescu MM Potretzke TA Microwave ablation for the treatment of hepatic adenomas. J Vasc Interv Radiol 2016;27:244–9. 26830938 10.1016/j.jvir.2015.09.021

[33] Shortell CK Schwartz SI Hepatic adenoma and focal nodular hyperplasia. Surg Gynecol Obstet 1991;173:426–31. 1658955

[34] Yoshidome H Mcmasters KM Edwards MJ Management issues regarding hepatic adenomatosis. Am Surg 1999;65:1070–6. 10551759

[35] Farges O Dokmak S Malignant transformation of liver adenoma: an analysis of the literature. Dig Surg 2010;27:32–8. 20357449 10.1159/000268405

